# A Diphenylalanine Based Pentapeptide with Fibrillating Self-Assembling Properties

**DOI:** 10.3390/pharmaceutics15020371

**Published:** 2023-01-21

**Authors:** Stefania-Claudia Jitaru, Andrei Neamtu, Gabi Drochioiu, Laura Darie-Ion, Iuliana Stoica, Brindusa-Alina Petre, Vasile-Robert Gradinaru

**Affiliations:** 1 Faculty of Chemistry, Alexandru Ioan Cuza University, 11 Carol I Bd., 700506 Iasi, Romania; 2 TRANSCEND—Regional Institute of Oncology, 700483 Iasi, Romania; 3 Department of Physiology, “Gr. T. Popa” University of Medicine and Pharmacy, 16 University, 700115 Iasi, Romania; 4 “Petru Poni” Institute of Macromolecular Chemistry, 41-A Grigore Ghica Voda Alley, 700487 Iasi, Romania

**Keywords:** pentapeptide, self-assembling, egg white lysozyme, MALDI-ToF, fluorescence, aggregation, AFM

## Abstract

Peptides and their related compounds can self-assemble into diverse nanostructures of different shapes and sizes in response to various stimuli such as pH, temperature or ionic strength. Here we report the synthesis and characterization of a lysozyme derived pentapeptide and its ability to build well-defined fibrillar structures. Lysozyme FESNF peptide fragment was synthesized by solid phase peptide synthesis using the Fmoc/t-Bu strategy, purified by analytical high-performance liquid chromatography (HPLC) and its molecular weight was confirmed by matrix-assisted laser desorption/ionization mass spectrometry (MALDI–MS). Spectroscopic features of this pentapeptide were investigated by UV-visible spectroscopy and fluorimetry showing the pattern of marginal phenylalanine residues within the peptide sequence. Self-assembling properties were determined using atomic force microscopy (AFM), aggregation index and thioflavin T assay (ThT). FESNF generating fibrillar structures observed by AFM and aggregation propensity were primarily influenced by pH conditions. Moreover, the experimental data were confirmed by molecular dynamics simulation studies. The obtained fibrils will be used next to explore their potential to act as support material for medical and cosmetic application.

## 1. Introduction

In the last few decades, self-assembling bioinspired molecules have gained significant interest due to their simple structure, biocompatibility and biodegradability. In particular, free or capped single amino acids or oligopeptides are renowned for these properties. Inspired by nature, many peptides have been designed to obtain diverse self-assembled structures based on well-known proteins. A wide range of applications were described so far for peptide self-assembled structures, such as drug delivery, tissue engineering, cell carriers biosensors and gene delivery [1,2,3].

Peptide biomimetics is an expanding research area that targets new synthetic materials with similar or unrevealed functions as compared to those of the widely investigated natural compounds.

The lysozyme is a low molecular weight enzyme that plays an important role in the innate immune response [4]. Hen white lysozyme has the capacity to assembly into fibrils under heating and acidic conditions [5]. Both fibrils and worm-like structures display antimicrobial and antifungal activities [6]. Lysozyme-like amyloid networks were successfully used as scaffold material for tissue engineering [7]. Molecular dynamics studies also support the lysozyme capacity to form beta-strands [8] and the self-assembly conformation of the lysozyme was shown to be a calcium dependent process [9]. An amyloid core region GILQINSRW named K peptide was found to be able to form aggregates at pH 4 [10]. Analogously, a recent study proposes to use peptides from hydrolyzed hen egg white lysozyme (HEWL) as a convenient source to prepare an amyloid hydrogel by lyophilization and resuspension at 37 °C and pH shifting [11]. Antioxidant and antimicrobial peptides derived from lysozyme enzymatic digestion were also reported [12,13]. Phenylalanine-, tyrosine- and tryptophan-containing peptides are easily detected in the 240–310 nm range [14] and easily quantified and studied by fluorescence [15] or used as platforms for assembly [16] or chemosensors [17]. Phenylalanine may act as relay amino acid in the electron transfer process through peptides [18]**,** which imprint antioxidant properties to some peptides [19]. Phenylalanine prefers homologues and heterologous interactions with tyrosine or tryptophane residues [20]. The assembly of uncapped, single- or double-capped phenylalanine homopeptides was recently revised. The self-assembly thermodynamics and kinetics is also dictated by N- and C-termini groups [21]. The number of aromatic residues, peptide concentration and incubation conditions are influencing peptides morphology and polymorphism [22]. The amphiphilic peptide palmitoyl-FFFEEE-COOH self-assembly of twisted helical ribbons was also reported [23]. The self-assembly kinetics of a model amyloid peptide YYKLVFF, having a core sequence derived from Aβ peptide, was also studied in solution by linear dichroism [24].

FTIR spectroscopy and differential scanning calorimetry were previously applied in order to study the thermal, aggregation and gelation capacity of the lysozyme in self-assembling conditions [25]. Waltz-DB 2.0 is a database of amyloid-like forming peptides that contains more than 1400 entries. According to this database, FESNFN is a peptide, among the other 40 phenylalanine based-hexapeptides, derived from hen egg white lysozyme primary sequence, which stands out by its amyloid propensity [26]. Analogously, by applying a Tango algorithm [27] prediction a possible amyloidosis conformation of this peptide was also suggested. According to our knowledge and literature search, experimental studies to elucidate the capacity of FESNFN hexapeptide to from fibril were not reported. However, the sequence FESNF was identified as (i) an epitope peptide selected by class MHC molecules [28], (ii) as part of tryptic peptide fragment ^34^FESNFNTQATNR^45^ for relative quantification in size exclusion chromatography coupled with tandem mass spectrometry [29] and (iii) as interacting moiety for NHS-aryl azido heterobifunctional cross-linker used to study large-scale protein–protein interactions by chemical cross-linking mass spectrometry [30]. This is the first time an experimental study indicates the possibility of long-distance phenyl moieties to be involved in self-assembly.

Inspired by these theoretical data, a pentapeptide NH_2_-FESNF-CO-NH_2_ has been selected in this study. Initially, we assumed that the presence of aromatic groups at both the N- and C-termini would cause π-π-stacking interactions and play a significant role in the self-assembling process. Since serine residue facilitate hydrogen bonding and glutamate moiety could be involved in electrostatic interaction, these residues are important in the native self-assembly process and scaffolds [31]. Herein, we present the synthesis, separation and characterization of C-terminal amidated FESNF peptide by spectroscopy and mass spectrometry. Atomic force microscopy (AFM), aggregation index and thioflavin (ThT) test assays and molecular dynamics simulations were used to investigate peptide self-assembling behavior.

## 2. Materials and Methods

### 2.1. Reagents

L-Phenylalanine was obtained from Fluka (Buchs, Switzerland). N,N-dimethylformamide (DMF), sodium acetate trihydrate, trifluoroacetic acid (TFA), dichloromethane (DCM), 2,5-dihydroxy-benzoic acid (DHB) and N-methyl morpholine (NMM) were purchased from Sigma-Aldrich (Steinheim, Germany). Rink amide resin (50–90 mesh, 0.51 mmol g^−1^) purchased from Sigma-Aldrich was used as a solid support. Acetic acid glacial and the amino acids protected at N-terminal with Fmoc group (9-fluorenylmethyloxycarbonyl) used in solid phase synthesis were purchased from Merck (Darmstadt, Germany) and piperazine—N,N′-bis (2-ethanesulfonic acid), PIPES buffer was from Carl Roth (Karlsruhe, Germany). Benzotriazol-1-yl-oxy-trispyrrolidinophosphonium-hexafluoro-phosphate (PyBOP) used as activator was purchased from NovaBiochem (Novabiochem, Merck KGaA, Darmstadt, Germany). Tris base (ULTROL^®^Grade) was obtained from the Calbiochem (EMD Chemicals, Inc., San Diego, CA, USA) and Thioflavin T from EMD (Millipore, Bedford, MA, USA). Acetonitrile (ACN) HPLC grade and piperidine were purchased from Merck (Darmstadt, Germany). All the solutions were prepared using deionized water (18.2 MΩ∙cm) produced by a Milli-Q system (Millipore, Bedford, MA, USA). All other reagents were used without further purification.

### 2.2. Peptide Synthesis

**Figure 1:**
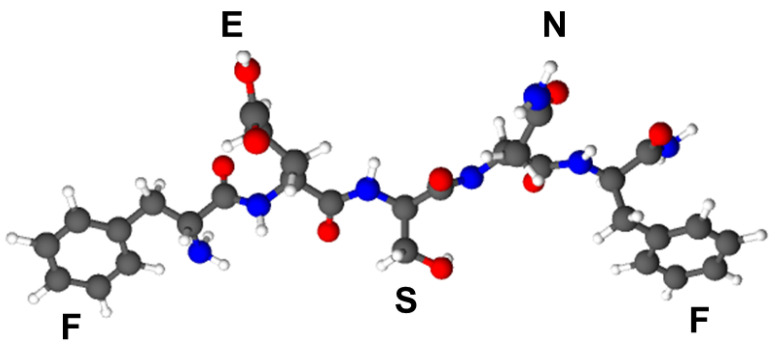
C-amidated pentapeptide (NH_2_-FESNF-CO-NH_2_) molecular structure (Balls and sticks model), where gray is carbon; blue is nitrogen; red is oxygen and white is hydrogen, where F-phenylalanine, E-glutamic acid, S-serine and N-asparagine.

The amidated peptide, NH_2_-FESNF-CO-NH_2_ (Figure 1) was synthesized by solid-phase peptide synthesis (SPPS) based on Fmoc/tBu strategy as previously described [32,33,34]. Rink amide resin (50–90 mesh, 0.51 mmol g^−1^) was used as a solid support. In summary, the protocol used was as follows: (i) Fmoc deprotection for 2, 2, 5 and 10 min using 20% piperidine in DMF; (ii) washing with DMF; (iii) coupling of Fmoc-amino acid/PyBOP/NMM in DMF for 50 min; (iv) washing with DMF; (v) second coupling of Fmoc-amino acid/PyBOP/NMM in DMF for 50 min. A 3-fold excess was used for PyBOP—coupling reagent and double coupling was performed using 5-fold excess followed by 3-fold excess of each sequential coupled amino acid. Bromophenol blue test has been used for monitoring the coupling progress [35]. After the synthesis was finished, a chemical solution containing TFA, triisopropylsilane and deionized water (95:2.5:2.5, *v*/*v*/*v*) was used to cleave the peptide from the resin at room temperature for 2.5 h. The crude product was afterwards precipitated with cold diethyl ether, solubilized in 5% aqueous acetic acid prior to freeze-drying using a lyophilizer from Martin Christ Alpha 1–2 LDplus (Martin Christ, Osterode am Harz, Germany) and stored at −20 °C until further use.

### 2.3. Reversed-Phase-High Performance Liquid Chromatography

The pentapeptide was purified using an HPLC Dionex UltiMate 3000 UHPLC system (Thermo Scientific, Waltham, MA, USA) equipped with a Diode Array Detector module. A Vydac RP-C18 column (5 µm silica, 250 mm × 4.6 mm, 300 Å pore size) from Waters (Milford, MA, USA) was used as stationary phase. A mixture of two eluents, A (0.1% TFA in bidistilled water) and B (0.1% TFA in ACN: bidistilled water, 80:20, *v*/*v*), was used as a mobile phase. A linear gradient elution, from 5 to 65% B within 30 min, with a flow rate of 1 mL/min was employed for HPLC separation. The peptide was detected by monitoring the typical peptide bond absorption at 215 and 220 nm, and characteristic band of phenylalanine moieties at 255 nm. Two main elution peaks were observed when crude peptide was analyzed by chromatography. Thus, beside the peptide elution peak observed at 11.64 min a byproduct having a retention time of 13.96 min was noticed. 

### 2.4. MALDI-ToF Mass Spectrometry

Mass spectrometry analysis was carried out using a Bruker Ultraflex MALDI ToF/ToF mass spectrometer. A 50 mg/mL DHB in 2:1 ACN: 0.1% TFA in MilliQ was used for peptide mapping. Consequently, the mixture was transferred and allowed to dry at RT. After samples cocrystallization (sample: matrix 1:1), on a 348-spot target plate, this was inserted into instrument. The following parameters were used to obtain MALDI-ToF MS spectra: positive ion mode, an acceleration voltage 20 kV, 140 ns delay, 40% grid voltage, low mass gate of 500 Da. The mass spectra acquisition was performed in a mass range of 600–3500 Da. A mixture of five peptides (ACTH, Angiotensin II, Bradykinin, Insulin, B-chain oxidized P14R and insulin) was used as an external mass calibrator. Each final mass spectrum was obtained as a result of 300 shots taken per each acquisition. In order to investigate peptide fragmentation LIFT cell in MALDI–ToF/ToF mass analyzer was used.

### 2.5. Spectrophotometric and Spectrofluorimetric Studies

UV-visible absorption spectra of purified pentapeptide were recorded in a 1 cm quartz cell using a Libra UV-visible spectrophotometer (Biochrom, Cambridge, UK) equipped with a Peltier thermostat cell holder maintained at 25 °C. Each spectrum was measured in triplicate using a scan speed of 1856 nm/min (scan step 1 nm), in the spectral range of 200–500 nm. All samples were dissolved and diluted in Tris 30 mM, pH 7.3. 

Index aggregation assay was performed at 37 °C for 150–180 min, according to Pignataro et al. [36] and Rajan et al. [37]. The peptide was dissolved in a corresponding buffer with final concentration of 1.0–2.0 mg/mL. Four different buffer solutions, Acetate 45 mM pH 6.0, Acetate 50 mM pH 7.0, Tris 40 mM pH 8.0 and Tris 50 mM pH 10.70 were used. Two solutions (HCl 10 mM and 0.1 mM) were used for acidic conditions. Each spectrum was recorded with a scan speed of 2649 nm/min (scan step 1 nm), in the spectral range of 200–400 nm at 37 °C. The highest aggregation index (Equation (1)), determined based on absorbance values recorded at both 258 and 350 nm, was reached in 20–30 min.

(1) $$AI=\frac{A_{350}}{\left(A_{X}-A_{350}\right)}*100$$

where *A*_350_ is absorbance at 350 nm and *A_X_* is the absorbance at 280 or 258 nm

Thioflavin T spectroscopic assay was performed using an λex = 450 nm and an λem = 482 nm. A stock solution of ThT was prepared in a freshly prepared NaPi 10 mM/NaCl 150 mM buffer pH 7. The ThT fluorescence was monitored in the presence of aggregate using a diluted dye solution (40 µL of 0.5 mM) at a final concentration of 40 µM using alpha-synuclein as positive control aggregating protein [38]. The increase of emissions was observed by adding a volume of 10 µL of peptide solution into the cuvette (final volume 500 µL) and recorded as mentioned above.

All fluorescence 2D and 3D spectra were recorded using a FP-8350 Spectrofluorometer (JASCO, Tokyo, Japan). The peptide solution was excited at different wavelengths to get emission spectra. Analogously, excitation spectra were taken at pH 5 in the range from 200 to 272 nm using an λem = 282 nm. The maxima of excitation spectra correspond to 258 nm from the absorbance. 

All measurements were carried out at 27 °C using a transparent quartz SUPRASIL^®^ cuvette (with 0.5 cm × 0.51 cm light path length, 1.7 mL volume, Hellma, Mulheim, Germany) and a cell support FMH-802 type. Two-dimensional emission or excitation spectra were recorded using a scan speed of 1000 nm/min, scan step 0.5 nm, 5 acquisition per sample; both excitation and emission slit were 5 nm. Emission spectra of peptapeptide were recorded in the range from 268 to 360 nm using an λex = 258 nm, corresponding to the maximum wavelength band of the compound. The peptide quenching experiments were executed in similar conditions following emission decrease at 283 nm. 

Three-dimensiona; fluorescence spectra of peptide were performed under the following conditions: excitation 200–350 nm; emission 210–360 nm; data interval 1 nm in excitation and emission; scanning rate 2000 nm min^−1^. Blank subtractions were considered for both 2D and 3D measurements and dilution correction were performed for 2D quenching data.

### 2.6. Atomic Force Microscopy 

The atomic force microscopy measurements were performed using a NTEGRA Scanning Force Microscope device (NT-MDT, Spectrum Instruments, Zelenograd, Moscow, Russia) equipped with a NSG10 cantilever (resonance frequency ν_res_ = 214 kHz) in atmospheric conditions at 23 degrees. Different scan sizes were imaged between 3 × 3 µm^2^ and 20 × 20 µm^2^ with a scanning frequency of 0.6 Hz; the scanning velocities were 3.6 and 24 µm/s, respectively. Pentapeptide stock solutions (10 mg/mL) were prepared in bidistilled water and further diluted 1:10 (*v*/*v*) in a 4.5% methanolic or buffer solution (ammonium acetate 50 mM pH 5.5, Tris 50 mM pH 7.4 or Tris 50 mM pH 8.2). A small volume (5 µL peptide solution) was transferred on a clean glass slide (10 × 10 mm) and incubated at room temperature for 48 h.

### 2.7. Replica Exchange and Coarse-Grain Molecular Dynamics Simulations

The structural models of the FENSF peptide were built in an extended conformation using the Schrödinger BioLuminate graphical environment (BioLuminate, Schrödinger, LLC, New York, NY, USA, 2021) [39]. The protonation states of the N-terminus and the aspartate residue in position 2 were chosen according to the pH of the simulation environment (acidic, neutral and slightly basic). The peptides were solvated in cubic simulation boxes so that there was a minimum distance of 9 Å between the peptides and the box boundaries. The peptides and ions were modelled based on the OPLS_2005 forcefield [40], while the TIP3P model was selected for water representation [41]. Replica exchange with solute scaling (REST2) [42] was used to efficiently sample the peptide conformational space using 6 replicas for the single peptide and 8 replicas for the di-peptide simulations respectively. All simulations, each 30 ns long, have been performed using the Desmond [43] molecular dynamics package at constant temperature (300 K) and pressure (1 atm) using the Nosé–Hoover chains thermostat [44] and Martyna–Tobias–Klein barostat [45]. Conformations resulted from the replica exchange simulations were clustered based on their root mean square deviation of atom position using the VMD software [46]. The dominant cluster was considered the one containing the largest number of structures. The coarse-grain (CG) simulations (0.8 µs long) were performed using the MARTINI [47] force field with the GROMACS 2019 software suite [48]. Visualization and coloring of molecular structures was done with the Schrödinger BioLuminate and VMD graphical solutions.

### 2.8. Data Analysis

The spectral 2D data were analyzed using the KaleidaGraph v.4.0 (Synergy Software, Reading, PA, USA) and 3D-surface emission plot was created in Excel version 2207 (Microsoft 365 package). Mass spectrometry data were analyzed using a Bruker’s Flex Analysis v.3.4 software (Bruker Daltonics, Bremen, Germany). The AFM data acquisition and analysis were performed using Nova 1.1.1.19891 and Image Analysis 3.5.0.20102 software developed by NT-DMT Spectrum Instruments.

## 3. Results and Discussion

### 3.1. Peptide Synthesis, Purification and MS Characterization

**Figure 2:**
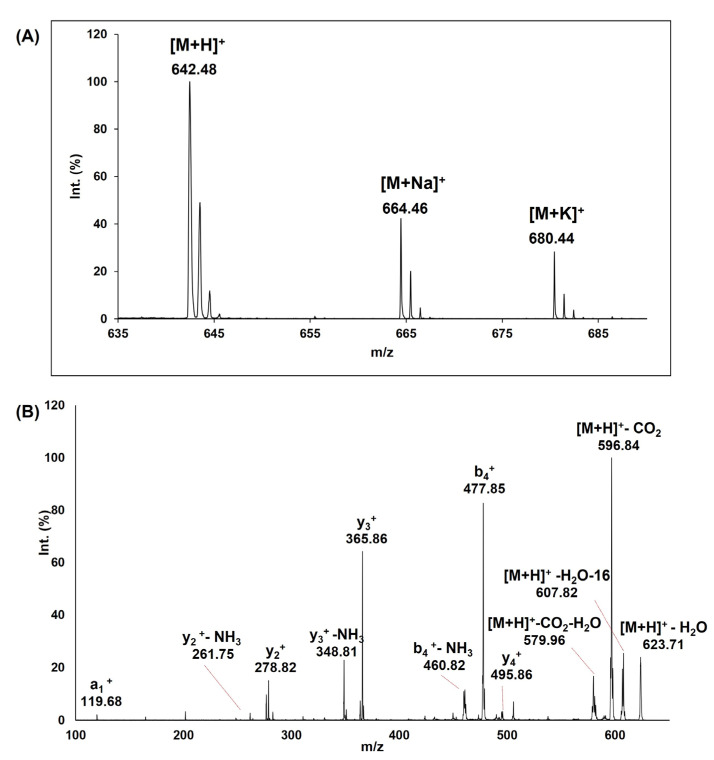
MALDI-ToF MS spectrum of FES peptide (**A**) and the MS/MS spectrum in LIFT mode of [M+H]^+^ ion (**B**).

The pentapeptide inspired from the hen egg white lysozyme primary structure was successfully synthesized by SPPS and purified by HPLC. Initially, the raw peptide was characterized by two signals in the separation profile (Figure S1, left). The initial peptide purity was estimated to be 79% and 77% based on the peak areas monitored at 215 and 220 nm. The by-product was eluted with a 2 min delay form the column. A peptide purity of 96% was attained after two purification steps (Figure S1, right). Both the peptide and by-product molecular weights were investigated by mass spectrometry using a MALDI-TOF MS and MALDI-ToF/ToF (Figure 2). The MALDI-ToF spectrum of FES peptide indicated an expected molecular weight of *m*/*z* 642.48 corresponding to [M+H]^+^. Moreover, the sodium, [M+Na]^+^ and potassium, [M+K]^+^ adducts were observed at *m*/*z* 664.46 and *m*/*z* 680.44, respectively (Figure 2A). The experimental values were compared with predicted values calculated using GPMAW software. MS/MS peptide sequence analysis was also used to confirm the primary structure of the FES peptide. As expected, the *m*/*z* 642.48 peptide parent ion fragmentation in LIFT mode led to several mono-charged b- and y-type ions and some dehydrated species (Figure 2B). Single positive charged peptide fragments were identified, such as y^2+^ (*m*/*z* 278.82), y^3+^ (*m*/*z* 365.86), b^4+^ (*m*/*z* 477.85) and y^4+^ (*m*/*z* 495.86). The peak intensity and cleavage preference is also influenced by amino acids situated near to the breakage site, which allow a different malleability for fragmentation depending on the structure of their side chain [49]. Additionally, the tandem mass spectrometric spectrum had several fragments, such as those resulted from the removal of a molecule of water, [M+H]^+^-H_2_O at *m*/*z* 623.71 fragment ion, ammonia, y^2+^-NH_3_ at *m*/*z* 261.75, y^3+^-NH_3_ at *m*/*z* 348.81, b^4+^-NH_3_ at *m*/*z* 460.82 fragment ion or carbon dioxide, [M+H]^+^-CO_2_ at *m*/*z* 460.82 fragment ion. The water loss can be attributed to glutamate and serine moiety and a 17 Da deviation from ammonia release assigned to the pentapeptide N-terminus or water release along with an isotopic shift [50]. On the other hand, the loss of carbon dioxide observed for the molecular ion and its dehydrated species originated from the decarboxylation of the glutamate moiety. 

Analogously, the second compound observed after synthesis was studied by MS and MS/MS analysis. A molecular weight shift of 106 Da was noticed for this by-product and was attributed to a different cleavage of the peptide from the resin. This cleavage pattern was also earlier reported for other peptides when rink amide was used as solid support and TFA as cleaving agent [51].

### 3.2. Spectral Properties Studies

The pentapeptide under the present study has two distinctive fingerprints due to phenylalanine residues at both the N- and C-terminal sites, which may predispose to a self-assembly behavior. The presence of these aromatic rings was investigated using UV-visible spectroscopy as well as fluorescence techniques in order to find another potential application for peptide-metal ion interactions.

**Figure 3:**
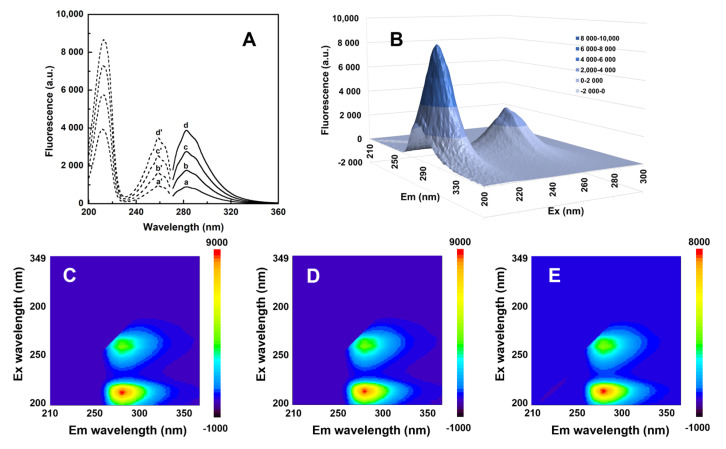
2D and 3D spectra of peptapeptide. Emission spectra (**A**) at four different peptide concentrations (39, 78, 132 and 176 µM—a, b, c and d; continuous lines) obtained at λ_ex_ 258 nm and the corresponding excitation spectra (a’, b’, c’ and d’; discontinuous lines) recorded at an λ_em_ 282 nm. Three-dimensional spectra of FESNF-NH_2_ peptide (156 µM, (**B**)) in acetate 50 mM pH 7.0. Contour map for pentapeptide collected at three different pH values (pH 5.0; 6.0, and 7.0 in (**C**–**E**)).

The UV-visible spectrum of pentapeptide was recorded in Tris buffer in order to establish the typical characteristic fingerprint of phenylalanine moieties. Thus, the highest peak noticed at 258 nm was accompanied by four spectral shoulders at 247, 252, 263 and 267 nm at both pH 7.3 and 8.0. A similar spectroscopic profile was noticed in PIPES buffer 30 mM pH 6.5 at a 200 µM peptide concentration (Figure S2). These findings are in the frame of previously reported data for phenylalanine containing peptides [52,53]. Another spectral band, characteristic of an amide group, was displayed at 203 nm (A 0.97) when a diluted peptide solution (41 µM) was analyzed at pH 7.3. Phenylalanine may act as a relay for long-distance electron transfer through the peptide structure [18] and its modest contribution to peptide intrinsic fluorescence is related to its low quantum yield [54]. Fluorescence studies also reveal specific features for peptides containing phenylalanine moieties. The emission spectrum of FES peptide in Tris 30 mM pH = 7.3 at excitation wavelengths of 258 nm displays a maximum at 282 nm and a shoulder at around 290 nm. A good linearity was noticed in the range of 25–250 µg/mL of pentapeptide at the emission wavelength of 282 nm (Figure S3, inset). Analogously, the emission peptide spectrum in sodium acetate 50 mM pH = 5.05 was measured and a similar profile was noticed. As expected, the excitation spectra display a maximum at 258 nm (Figure 3A).

Three-dimensional fluorescence spectra were also investigated at pH 5, 6 or 7 in 40 mM acetate buffer at 27 °C. Peptide alone (156 µM) displays 2 peaks centered at 213 and 283 nm (Figure 3B–E). These bands are characteristic to phenylalanine residues and not to peptide backbone [55].

### 3.3. Peptide Aggregation Studies

Peptides’ and proteins’ physical stability is usually influenced by a number of factors. Amino acid sequence, peptide net charge and its concentration might influence the peptide’s ability to form aggregates [56]. A recent guide focused on the investigation of protein aggregation was recently published. Some peptide aggregation propensity prediction tools and various experimental methods are available to study aggregating species including their shape and size [57]. 

**Figure 4:**
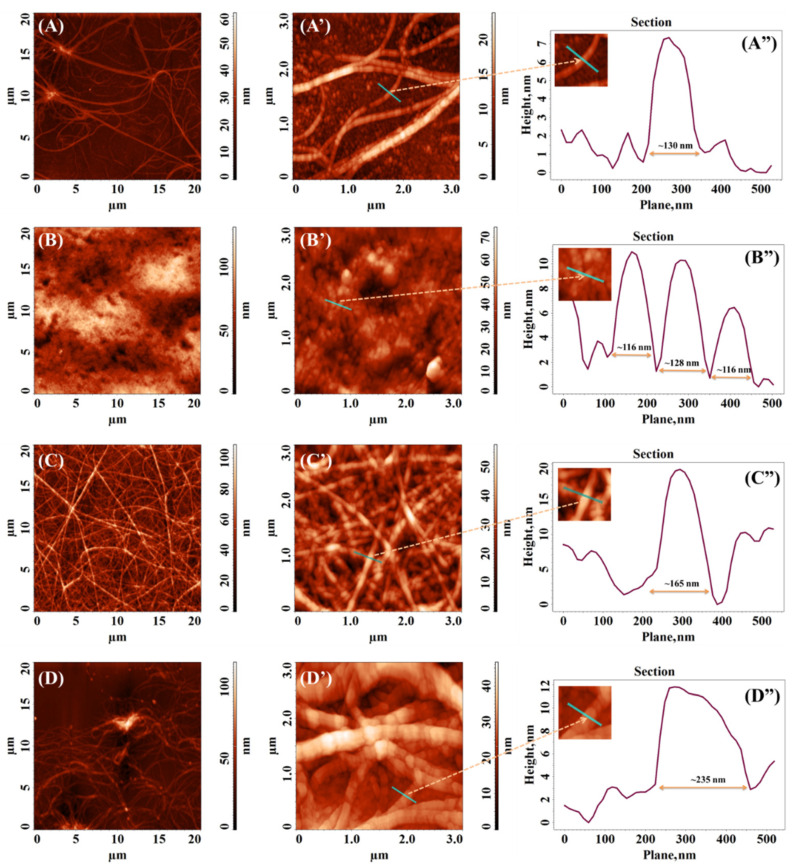
Two-dimensional (2D) AFM images collected on 20 × 20 µm^2^ and 3 × 3 µm^2^ areas and the corresponding cross-section profiles of the samples obtained from various FESNF-CO-NH_2_ solutions (1 mg/mL) in (**A**,**A’**,**A”**) ammonium acetate 45 mM pH 5.5; (**B**,**B’**,**B”**) Tris 45 mM pH 7.4; (**C**,**C’**,**C”**) Tris 45 mM pH 8.2; and (**D**,**D’**,**D”**) 4.5% methanolic solution.

The aggregation index is an important parameter that could indicate a peptide’s ability to form aggregates in solution, usually when the aggregation index (AI) exceeds the value of three. Phenylalanine alone has the capacity to form fibrils in PBS at 37 °C [58]. Moreover, the presence of phenylalanine residues in hexapeptides in cross-β aggregates and amyloid-like species was previously reported in the literature [27] and aggregation propensity is strongly influenced by the number of phenylalanine residues present in the molecule [59]. Additionally, the asparagine or glutamine could play an important role in seeding peptide and protein aggregation [60,61]. As expected, the obtained aggregation index was higher than 40 at pH 6.00, 8.00 and 10.7 when A_280_ was considered. These values, obtained applying Equation (1), could be particularly assigned only for protein aggregation studies. Conversely, more appropriate results were obtained for our pentapeptide when small AI values (less than 3) were calculated based on A_258_ and A_350_. A dynamic aggregation profile is shown in Figure 4. The highest AI (2.8) was obtained at pH 6 after 20 min incubation at 37 °C. Similarly, pentapeptide aggregation was studied at pH 2. Interestingly, in these conditions the plateau was reached after 30–40 min when a solution of 2 mg/mL peptide was used. The aggregation index was also determined as a function of pH after 30 min incubation at 37 °C. The bell-shape profile (Figure S4 inset) clearly suggests that the peptides need a slightly acidic environment to promote a propensity for aggregation.

Phenylalanine forms aggregates and ThT fluorescence intensity increases due to the entrapment of the dye in these self-assembled structures [62]. The peptide aggregation capacity was also investigated using the ThT assay. In this assay, the response could be also influenced by FESNF total charge. Thus, after an incubation time at 50 degrees for 145 ha, an 18% fluorescence increase was noticed at pH 8 (Figure S5). This electrostatic interaction of positively charged ThT with the investigated partially negative peptide could enhance the response in the dye fluorescence signal. This prominent role of electrostatic interactions between fibrils and ThT was demonstrated as a major parameter affecting fluorescence response [63].

Thus, after optimizing the pH and ionic strength the pentapeptide self-assembly potential was experimentally revealed by aggregation index studies providing new possibilities for developing higher ordered structures using small molecules, such as peptides, peptide conjugates and peptoids. To avoid misinterpretation of experimental data, a careful analysis should be performed. The ThT assay shows a slight aggregation tendency; however, this method is costly, time consuming and difficult to be applied for short peptides. 

### 3.4. Atomic Force Microscopy (AFM)

The morphology of the aggregated species derived from FESNF-CO-NH_2_ under different solution media were analyzed using AFM and presented in Figure 4.

Both ammonium acetate pH 5.5 and methanolic solutions (Figure 4A,A’,D,D’, respectively) appear to induce the clumping of fibrils. Although, this effect is more noticeable in the case of Figure 4D,D’,D”, which confirms once again that short-chain alcohols induce retardation on fibril formation as previously described in another study [64]. Moreover, methanol has been reported to substantially change the prevailing morphology by significantly decreasing the drying time [65]. The fibrils observed on the sample derived from the ammonium acetate pH 5.5 appear similar to a real neural network. In this case, the average width of the fibrils is approximately 130 nm, compared to that of the fibrils obtained for the sample from methanolic solution, where the average width is higher, around 235 nm, as can be seen from the measurements based on the cross-section profiles from Figure 4A”,D”. 

The calculated isoelectric point (pI) of FESNF-CO-NH_2_ peptide was 6.99, according to BACHEM Peptide Calculator tool [66]. At pH 7.4 (Figure 4B,B’), under physiological conditions and near the pH value of pI, peptide fibrillation is completely absent. Dense clusters were formed, with dimensions ranging between 116 and 128 nm, according to the cross-section profile (Figure 4B”). This is a similar behavior exhibited by bovine insulin when exposed to the corresponding pH value to its pI [64]. In contrast, fibrillation of Alzheimer amyloid peptides tends to be favored when the net electrical charge is zero due to pH conditions [67].

However, at pH 8.2 when negative charges outnumber positive charges, the peptide is negatively charged. The salts could trigger peptide fibrillation [68]. The glutamate deprotonation could discourage intra and intermolecular H-bond interactions triggered by glutamate, serine and asparagine sidechains from the peptide main core and additionally enhances intermolecular interactions via π-π staking. In these conditions, the pentapeptide self-assembles near to the pKa value of Tris buffer into long homogenous and distinct nanofibrils (Figure 4C,C’) with an average width of about 165 nm (see the section image from Figure 4C”). Therefore, we should consider contributions of both partially dissociated-NH_2_ groups from both peptide N-terminus and buffer. The fibrils seem to be the result of multiple globular structures stacked together, as observed in the zoomed image placed in the corner of the height profile (Figure 4C”). These results are framed with our recent study when a sensor for the detection of verbascoside in various olive oils by immobilizing FESNF peptide using glutaraldehyde as a crosslinking agent on screen-printed carbon electrodes that was modified with graphene oxide. A peptide fibrillary network was also observed on the surface [69]. Thus, Tris could act as a trigger to form more evolved 3D structures. A regular dense network of fibers having small diameters (4–5 nm) was noticed for the FEFEFKFK peptide by AFM and TEM [70].

As a result, a clear peptide assembly capacity was demonstrated by AFM, indicating that aggregated species grow in a pH-dependent manner. The aggregation index studies are in the frame with AFM investigations demonstrating the pH responsiveness of self-assembling. 

### 3.5. Replica Exchange Molecular Dynamics Simulations

Molecular simulations were used in order to extract information at the atomic scale on the conformational preference of the ‘FENSF’ peptide, which could be related to experimental results. In general, peptides do not adopt a single conformation in solution but rather they exist as an ensemble of conformations. However, if the peptides contain residues that participate in strong salt bridge interactions or are highly hydrophobic in nature, this may bias the conformational landscape towards local basins with reduced flexibility [71]. Constructing the ensemble of conformations of even small peptides is a difficult task to be achieved by conventional molecular dynamics simulations [72] due to the trapping of the system in local free energy minimum conformations for long periods of time. Thus, we used a replica exchange with solute tempering (REST2) [42] simulations to improve the sampling of the peptide conformational space.

**Figure 5:**
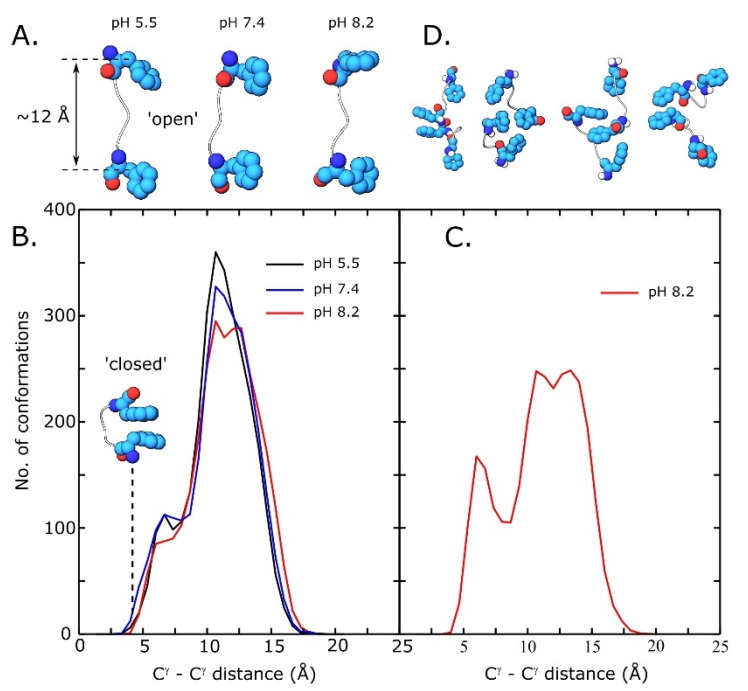
Conformational analysis from single (**A**,**B**) and di-peptide (**C**,**D**) peptide REST2 simulations. (**A**)—dominant conformation extracted from cluster analysis at acidic, neutral and slightly basic pH; (**B**)—histogram of end-to-end C^γ^–C^γ^ carbon atom distance distributions of terminal Phe residues in single peptide simualtions; (**C**)—histogram of end-to-end C^γ^–C^γ^ distance distributions in di-peptide simulations; and (**D**)—conformations of peptide dimers extracted from di-peptide simulations.

We were mainly interested in following whether the Phe residues present at the peptide ends may participate in intramolecular π-π interactions. We postulate that the preference for such a conformation would impair hydrophobic-driven growth of large aggregates due to steric clashes between many adjacent molecules. The results of the isolated peptide replica exchange simulations revealed that regardless of the pH value the peptides adopt a preferred extended (“open”) conformation with the Phe aromatic side chains placed distantly from each other (Figure 5A). Conformations involving intramolecular π-π stacking (“closed” conformations) are very rarely visited as it can be seen from the histograms of the end-to-end distance between C^γ^–C^γ’^ carbon atoms of Phe1 and Phe5, respectively (Figure 5B).

When two peptides were simulated together, intermolecular interactions involving terminal Phe side chains dominated the conformation of the binary ensemble (Figure 5D). These interactions were possible as the terminal Phe side chains were free to participate in π-π stacking interactions between two adjacent molecules. Interestingly, the intramolecular conformational preference of each peptide was modified by the presence of the interaction partner. The histogram of the end-to-end C^γ^–C^γ^ distance now shows a second peak at ~6 Å (Figure 5C) compared with only one dominant peak centered at ~12 Å for single peptide simulations. This increased tendency towards the ‘closed’ conformation is stabilized by simultaneous intra and intermolecular π-π interactions (Figure 5D). These results show that the ‘open’ conformation presents an advantage in terms of aggregation, leaving free endings available for the hydrophobic-driven aggregate growth.

**Figure 6:**
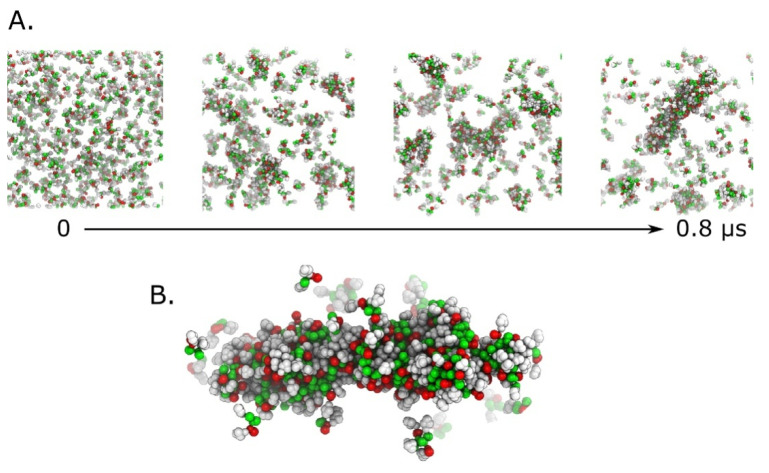
Coarse grain (CG) simulation of ‘FENSF’ peptide self-assembly at slightly basic pH. (**A**)—time series of peptide aggregates growth; (**B**)—detail of a large fibrillar aggregate formed after ~0.8 µs.

To further evaluate the tendency of the ‘FENSF’ peptide to aggregate we conducted coarse grain (CG) molecular simulations. In this type of calculation, chemical groups composed of several atoms are represented as a single interaction center. This allows the use of a much larger number of molecules and reaching time scales of at least an order of magnitude higher. Figure 6A shows the results of a 0.8 µs CG simulation including 400 FENSF peptides at pH 5.5. In the initial configuration, the peptide molecules were randomly placed in the simulation box. During the simulation, they self-organize into increasingly larger aggregates and finally form a fibrillar structure detailed in Figure 6B.

Concisely, coarse grain (CG) simulation of the ‘FENSF’ peptide self-assembly in slightly basic conditions reveals the peptide tendency to form fibril-like structures. The theoretical simulation data are consistent with AFM data and π-π stacking interactions and the open-state conformation might play an important role in the final 3D architecture.

## 4. Conclusions

The self-assembly of short new peptides with phenylalanine residues separated by three to five amino acids could be tailored to design new biomaterials with diverse applications. In this work, a FESNF peptide from a hen egg lysozyme was selected, synthesized, and characterized, and its propensity for fibrillation was investigated. AFM data clearly show that FESNF pentapeptide displays the ability to self-assembly into ordered and high dense fibrils in slightly alkaline condition, while their length decreased in methanolic solution. This fibrillation is most likely caused by interaction between the two aromatic groups that flank the peptide ends as confirmed by simulated data.

In terms of potential applications, the results presented in this study are promising and could be expanded by exploiting the fibrillation capacity of short new aromatic peptides and their coresponding mutants in the development of a scaffold for cell cultures and designing new drug delivery systems or peptide-based biosensors. When compared to native protein, the FESNF peptide self-assembly process was more challenging to evaluate using conventional spectroscopic approaches or all-atom strategies. 

These data imply that self-assembly properties of short phenylalanine peptides containing few cleavage sites in biogenic conditions should be multidisciplinary approached, beginning with a rational design, novel synthesis strategies, molecular simulation and spectral and modern imaging methods.

## Data Availability

Not applicable.

## Supplementary Materials

The following supporting information can be downloaded at: https://www.mdpi.com/article/10.3390/pharmaceutics15020371/s1. Figure S1: Chromatograms of crude and purified FESNF peptide. Figure S2: UV-visible spectra of pentapeptide in Tris 30 mM pH 7.3 (a and b, 826 µM and 41 µM) and acetate 40 mM pH 7.0 (600 µM, spectrum c, dotted). Figure S3: Emission spectra of pentapeptide (1, 2,3 and 4 at 26; 80; 132 and 238 µg/mL) in Tris 30 mM pH 7.3. The inset: fluorescence intensity as function of peptide concentration. Figure S4: Normalized aggregation index (AI) calculated for both 258 nm and 280 nm values. The maximum values for AI were 2.8 (full dots) and 44 (empty dots), respectively. Inset: aggregation index (AI) variation as function of pH values. Figure S5: Fluorescence emission (λex 440 nm and λem 482 nm) of pentapeptide with 40 µM ThT at pH 8.0 (black squares), pH 7.0 (black triangles) and pH 2.0 (open circles) as a function of incubation time at 50 °C.
