# Supplementary material for: A Diphenylalanine Based Pentapeptide with Fibrillating Self-Assembling Properties
Source: Pharmaceutics. 2023 Jan 21;15(2):371. doi: 10.3390/pharmaceutics15020371 (PMC9966497; doi:10.3390/pharmaceutics15020371)
Supplement: Supplementary file 1 [file pharmaceutics-15-00371-s001.zip › pharmaceutics-2097080-supplementary.pdf]

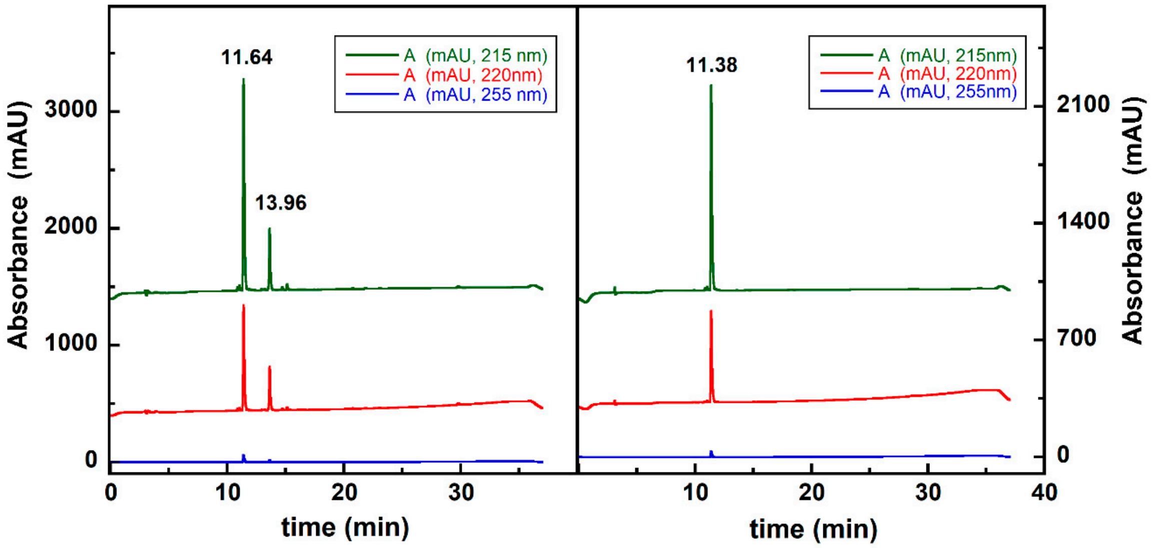

Figure S1. Chromatograms of crude and purified FESNF peptide.

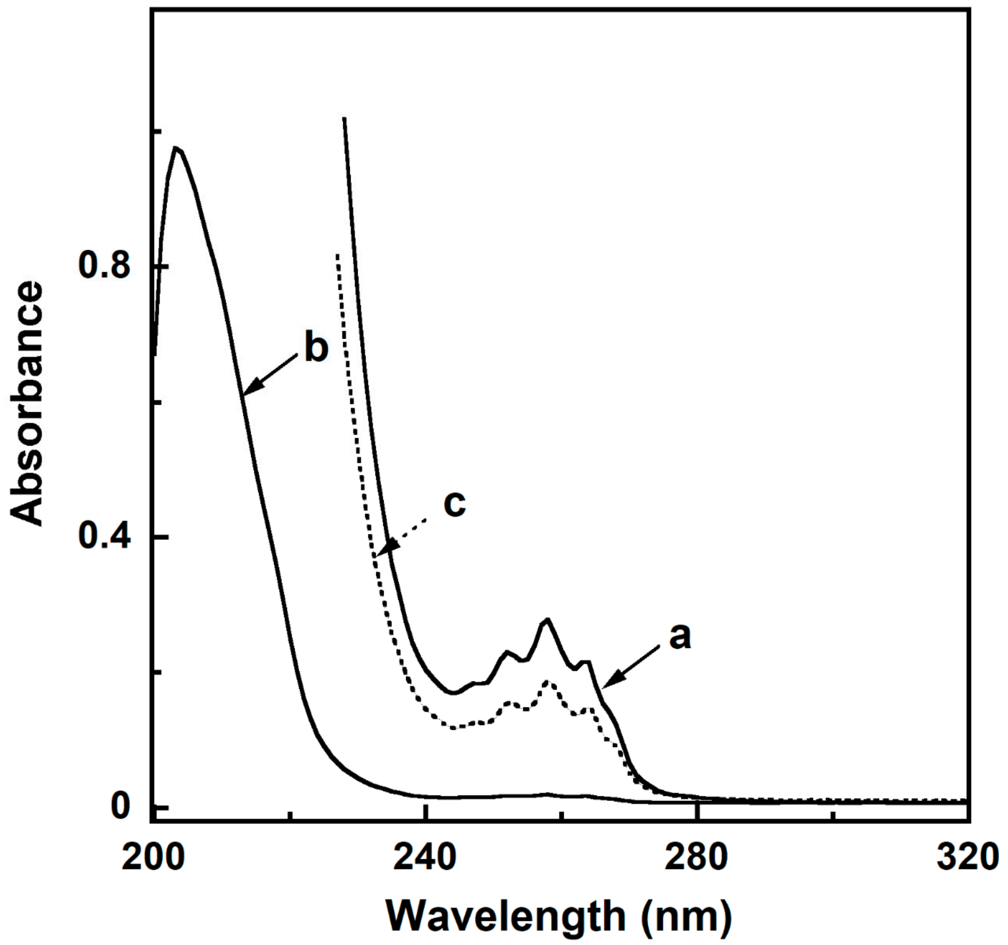

Figure S2. UV-Vis spectra of pentapeptide in Tris 30 mM pH 7.3 (a and b, 826 μM and 41 μM) and acetate 40 mM pH 7.0 (600 μM, spectrum c, dotted)

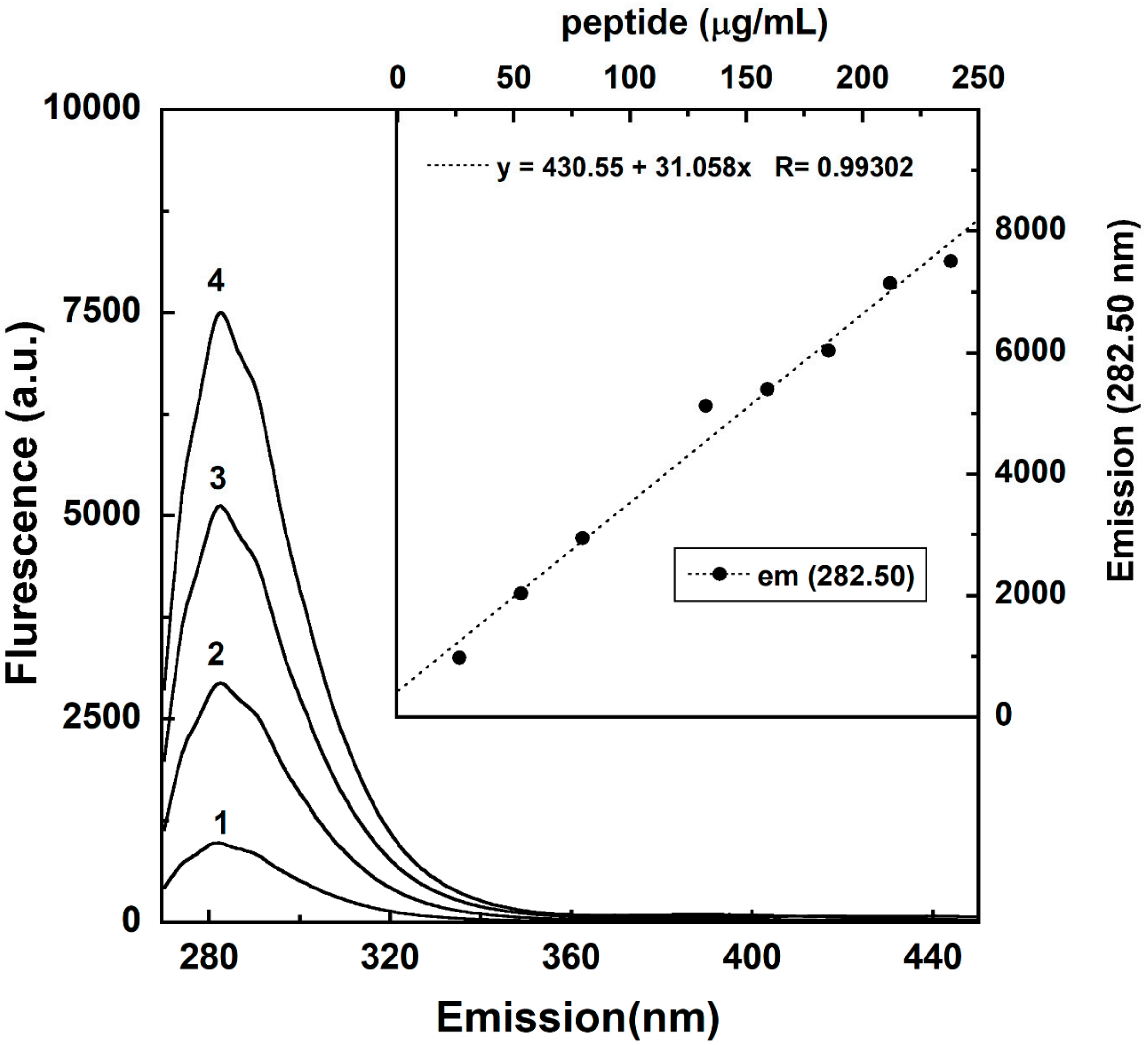

Figure S3 Emission spectra of pentapeptide (1, 2, 3 and 4 at 26; 80; 132 and 238 μg/mL) in Tris 30 mM pH 7.3. The inset: fluorescence intensity as function of peptide concentration.

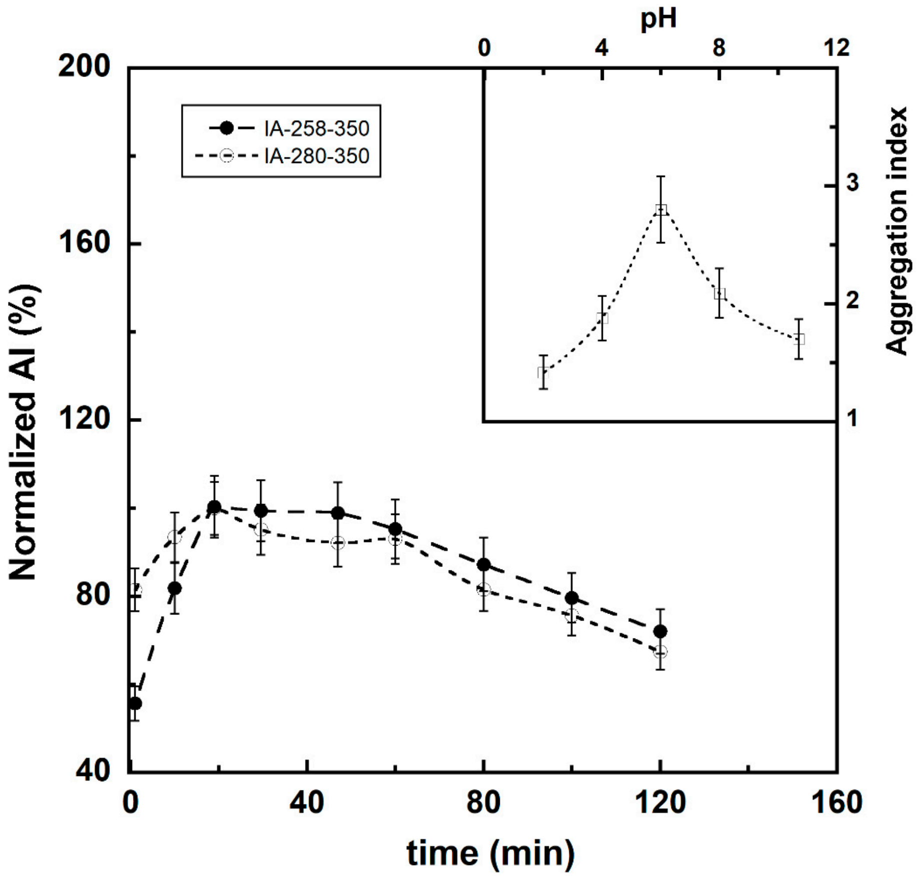

Figure S4. Normalized aggregation index (AI) calculated for both 258 nm and 280 nm values. The maximum values for AI were 2.8 (full dots) 44 (empty dots), respectively. *Inset:* aggregation index (AI) variation as function of pH values.

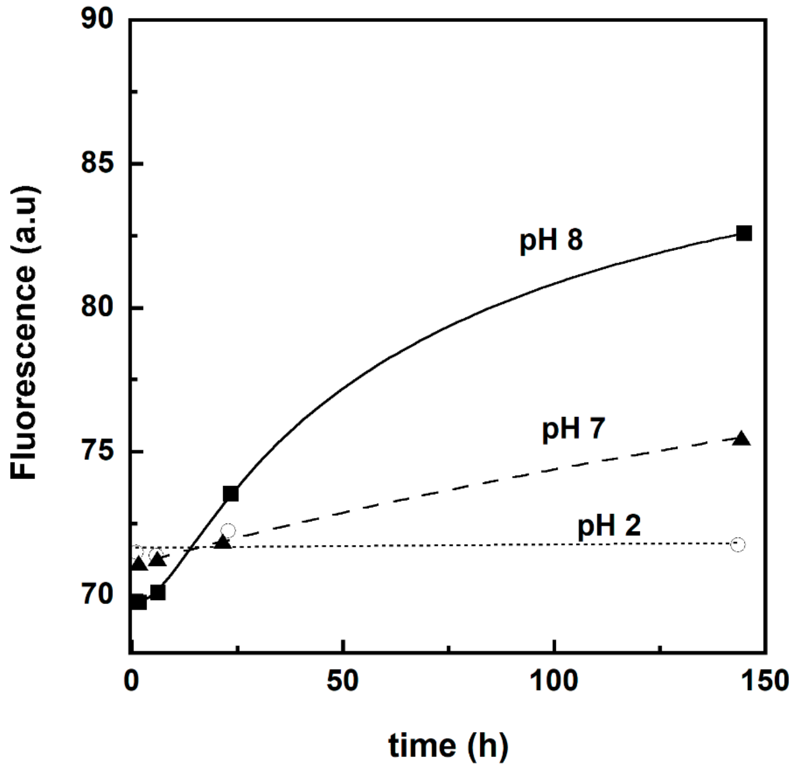

Figure S5. Fluorescence emission (λ_{ex} 440 nm and λ_{em} 482 nm) of pentapeptide with 40 μM ThT at pH 8.0 (black squares), pH 7.0 (black triangles) and pH 2.0 (open circles) as a function of incubation time at 50 ^{0}C.
